# Measuring children’s emotional and behavioural problems: are SDQ parent reports from native and immigrant parents comparable?

**DOI:** 10.1186/s13034-019-0306-z

**Published:** 2019-11-28

**Authors:** Ronja A. Runge, Renate Soellner

**Affiliations:** Institut für Psychologie, Stiftung Universität Hildesheim, Universitätsplatz 1, 31141 Hildesheim, Germany

**Keywords:** SDQ, Parent report, Confirmatory factor analysis, Immigrant, Mental health, Children, Measurement invariance

## Abstract

**Background:**

The number of immigrants worldwide is growing and migration might be a risk factor for the mental health of children. A reliable instrument is needed to measure immigrants' childrens mental health. The aim of the study was to test the measurement invariance of the parent version of the Strengths and Difficulties Questionnaire (SDQ) between German native, Turkish origin and Russian origin immigrant parents in Germany. The SDQ is one of the most frequently used screening instruments for mental health disorders in children.

**Methods:**

Differential Item Functioning (DIF) was tested in samples matched by socio-economic status, age and gender of the child. A logistic regression/item response theory hybrid method and a multiple indicators- multiple causes model (MIMIC) was used to test for DIF. Multi Group Confirmatory Factor analysis (MGCFA) was used to test for configural invariance. Parent reports of 10610 German native, 534 Russian origin and 668 Turkish origin parents of children aged 3–17 years were analysed.

**Results:**

DIF items were found in both groups and with both methods. We did not find an adequate fit of the original five factor model of the SDQ for the Turkish origin group, but for the Russian origin group. An analysis of functional equivalence indicated that the SDQ is equally useful for the screening of mental health disorders in all three groups.

**Conclusion:**

Using the SDQ in order to compare the parent reports of native and immigrant parents should be done cautiously. Thus, the use of the SDQ in epidemiological studies and for prevention planning is questionable. However, the SDQ turns out to be a valid instrument for screening purposes in parents of native and immigrant children.

## Background

The number of international immigrants increases rapidly worldwide, from 1990 to 2017 it rose by 69% [1]. Germany hosted the third largest numbers of immigrants all over the world in 2017, 16.1% of the German population migrated from another country. In the age group of children under five years the proportion of children of immigrants accounted for 39% in 2017 [2]. Monitoring the mental health of those children is a societal task, keeping in mind that being an immigrant might be a risk factor for children’s mental condition [3]. In order to achieve high quality data, a reliable instrument for measuring mental health problems is needed, measuring the same underlying constructs and thus providing comparable scores between native children and children of immigrants, to assess the need for specific preventive interventions and treatment programs [4].

For younger children in general parent reports are used. Immigrant parents however might be rooted in the culture of their country of origin, which might affect the way they report about their children. This could lead to non-comparable parent reports between groups of different cultural origin. Differences in reporting could be due to specific response styles (tendencies to agree or disagree to items of a questionnaire) in different countries [5], the use of different reference groups when evaluating oneself [6] or unalike societal norms, which are associated with different expectations how a child should behave or when certain developmental steps should happen. Different degrees of social desirability of a behaviour could result in different probabilities that problematic behaviour of one’s own child is reported [7–11].

In Germany, the largest immigrant groups are from Turkey, Poland and Russia [2]. In the current study, we will focus on Turkish and Russian immigrants. The majority of the Russian immigrants are ethnic Germans who came to Germany after the collapse of the Soviet Union (as *Spätaussiedler*) and got the German citizenship after arrival. Most people of Turkish origin living in Germany are work immigrants (or their descendants and family members), who came during the economy boom in Germany between 1950s and 1970s (as *guest workers*). Turkish citizens are the biggest group of people without a German citizenship living in Germany [2, 12].

Harzing [5] found differences in response styles between people in Germany, Turkey and Russia: Disacquiescence, the tendency to disagree with an item, was more often found in Russia compared to Germany and acquiescence, the tendency to agree with an item, was more often found in Turkey than in Germany. If these response styles are still prevailing in immigrants from these countries, scale values might be biased.

To date, some research about developmental expectations and parenting values in Turkish immigrants in Germany and less about Russian immigrants was conducted. Turkish immigrant parents in Germany expected their children to have close relations within the family, to support the family and to be obedient and well-mannered more often than German native parents and they were less likely to value autonomy or self-control [13–15]. Parents from Russia expected their children to be obedient more often than German parents [14].

In the current study we want to investigate if, despite the potential differences in parental response styles and in societal norms mentioned above, a widely used instrument for the screening of mental health, the Strength and Difficulties questionnaire by Goodman (SDQ; [16]) provides comparable scores when answered by German native parents and parents of Turkish or Russian origin. The SDQ was developed in the United Kingdom, but is in use worldwide [17]. Several studies used the SDQ to compare the mental health of native and immigrants's children in Germany [18–21] and in other western countries [22–24]. Goodman [16] proposed a five factor structure for his questionnaire (representing the subscales hyperactivity, peer problems, conduct problems, emotional problems and prosocial behaviour), each subscale of which contains five items. The factor structure and the psychometric characteristics of the questionnaire have been mostly investigated separately for different countries (for reviews see e.g. [25–28]). A lot of these studies confirm the five factor structure, others support a three factor solution (internalizing problems behaviour, externalizing problem behaviour and prosocial behaviour, as first order factors, e.g. [29] or second order factors e.g. [30]), or other solutions e.g. [31]. Studies questioning the cross-cultural validity of the parent-version of the SDQ draw inconsistent conclusions. While Stone et al. [32] found satisfactory internal consistency, test–retest reliability, and inter-rater agreement for the parent version of the SDQ for different countries in their review, Kersten et al. [25] reported a lack of evidence for cross-cultural validity and Stevanovic et al. [33] conclude, that there is only weak evidence for cross-cultural validity of the SDQ parent version. Apart from the factor structure, people in different countries or different ethnic groups within one country do not rate the same amount of behaviour reported as similarly problematic, show different SDQ sum scores and the correlations between SDQ scores and the results of mental disorder diagnostic interviews vary in different countries [34–40]. Concerning the most relevant countries of origin of immigrants in Germany, Turkey and Russia, there is only limited research about the validity of the SDQ parent version. Güvenir et al. [41] reported a high internal consistency (except for the peer problem scale) and a good convergent and discriminative validity of the SDQ in Turkey but did not test the fitting of the proposed five-factor structure. Stevanovic et al. [42] could not confirm the five-factor structure for adolescents’ self-reports in Turkey. Husky et al. [43] found that the SDQ score predicted mental health disorders equally well in Turkey and Germany, but also found low internal consistency for the peer problems subscale in the Turkish sample. In Russia, adolescents’ SDQ self-reports also showed inadequate psychometric characteristics [44]. Goodman et al. [37] investigated the comparability of the parent version of the SDQ in Britain, Russia and other countries and concluded that cross-national differences in SDQ indicators do not necessarily reflect comparable differences in disorder rates. In Russia, the SDQ total difficulties score led to an overestimation of disorder prevalence. A study investigating the factor-structure of the SDQ parent version in Russia does not seem to exist so far.

Few studies tested the comparability of SDQ results between ethnic groups within one country. Zwirs et al. [40] compared the factor structure of the SDQ rated by Dutch and Surinamese teachers and found measurement invariance, Richter et al. [45] explored self-reports of ethnic Norwegian and ethnic minority adolescents in Norway and found a good fit of the five-factor model in ethnic Norwegian adolescents and an acceptable fit in ethnic minority subsamples, but no measurement invariance between the samples. To our knowledge, only one study so far has investigated measurement invariance of the parent version of the SDQ in native and immigrant parents: Goodman et al. [46] compared a British Indian with a native British sample and found strict invariance in the parent version when excluding the prosocial scale from the analysis.

In the current study we aim to test the measurement invariance, and therefore the comparability, of the SDQ parent version between native German parents and parents of Russian and Turkish origin. We also were interested if the SDQ has the same predictive value for mental health disorders in these three groups, thus testing the SDQ’s functional equivalence.

## Method

### Data source

We used data from two waves of the German Health Interview and Examination Survey for Children and Adolescents (KiGGS), a nationwide survey in Germany, representative for children and adolescents, conducted by the Robert Koch Institute (RKI). For the analysis of measurement invariance, we used the data from the first survey wave, conducted from 2003 to 2006 [47]. To increase sample size, data from second survey wave (2009–2012, [48]) was added (respondents, who did not take part in the first wave). Several steps were taken to ensure a representative sample of migrants in the first wave’s sample: migrants were oversampled, invitation and interview material was translated in six languages (including Turkish and Russian), non-responders were contacted by phone or visited to reduce worries and fears and interviewers were culturally trained [49]. In the second wave, the extra steps mentioned above were not taken, resulting in a non-representative sample of migrants [48]. For the analysis of functional equivalence, cross-sectional (within the 1. study wave) and longitudinal data was used.

### Measures

#### SDQ

Children’s emotional and behavioural problems were assessed with the parent-version of the Strengths and Difficulties questionnaire [16], a short questionnaire measuring behavioural strengths and weaknesses of children or adolescents aged 4–17 years. Five subscales (hyperactivity, peer relationship problems, conduct problems, emotional problems and prosocial behaviour) are proposed, each of them consisting of five items. Each item can be answered with “not true” (0) “somewhat true” (1) or “certainly true” (2). While most items describe problematic behaviour and are therefore phrased negatively, some items are formulated positively.

#### Socioeconomic status (SES)

An overall SES measure was used, containing information about income, education and employment status. Children in the lowest SES score quintile are defined as “low SES”, in the second lowest to second highest quintile as “medium SES” and in the highest quintile as “high SES”. See [50] for a more detailed description.

#### Immigrant group

The interview partner was allocated to the group of persons of Russian/Turkish origin if he or she was born in Russia/Turkey, had the Russian/Turkish citizenship or stated to speak primarily Russian/Turkish at home. If mothers and fathers were interviewed together, they were allocated to the groups if both of them met one of the characteristics mentioned. *N *= 2 couples were excluded, because they answered the interview together but only one of them was of Turkish/Russian origin.

#### Functional equivalence measures

We used the sum score of the short form of the Patient Health Questionnaire, the PHQ-8 [51] as indicator for depression. Parents were asked, if the child was ever diagnosed with Attention Deficit Hyperactivity Disorder (ADHD) and if the child was ever diagnosed with any mental health disorder. Additionally, they were asked if the child has had contact to a psychiatrist, psychologist or psychotherapist in the last 12 months. Answers for diagnoses and contact were dichotomous (yes/no).

### Statistical analysis

To examine differences in response behaviour due to cultural origin, we wanted to minimize the influence of other factors potentially causing bias. Therefore, for testing measurement invariance, we draw two subsamples from the German native parents group: One was matched in SES, child’s age and gender to the Russian origin group (matched sample 1), the other to the Turkish origin group (matched sample 2). This was done using the IBM Statistical Package of Social Sciences (SPSS) version 25.0 for Windows.

Measurement invariance was examined by testing for Differential Item Functioning (DIF) in the subscales and the total difficulties scale and by checking for equivalence of the factor structure. DIF was performed by using the *lordif* package in R, which uses a logistic regression/Item Response Theory (IRT) hybrid DIF detection method, and by using McFaddens pseudo *R*2 > 0.02 as detection criterion [52]. To check the stability of results, we also used the multiple indicators, multiple causes (MIMIC) confirmatory factor analysis method with scale purification as proposed by Wang, Shih and Yang [53] within the *lavaan* package in *R* [54]. The MIMIC approach tests for uniform DIF. As recommended for ordinal data with medium sample sizes [55] diagonally weighted least squares (DWLS) were used to estimate the model parameters. Robust test statistics are reported. To evaluate the size of DIF effects in the MIMIC framework, a MIMIC effect size (MIMIC-ES) as proposed by Jin et al. [56] was calculated, with 0.3 indicating a small, 0.5 indicating a medium and 0.7 indicating a large effect. Additionally, Multi Group Confirmatory Factor Analysis (MGCFA) in lavaan was performed to examine equivalence of the factor structure with and without items flagged for DIF in the previous step. Model parameters in the MGCFA were also estimated using DWLS. In order to compare results with other studies using MGCFA to test for measurement invariance [e.g. 31, 33, 45], we additionally tested measurement invariance within this approach. We followed the process recommended by Hirschfeld and Von Brachel [57] with first establishing a configural model, second testing for configural equivalence (same loadings are significant across groups), third testing for weak/metric equivalence (loadings are constrained to be equal) and fourth testing for strong/scalar invariance (intercepts are constrained to be equal). We used *χ*^2^, the Comparative Fit Index (CFI) and the Root Mean Square Error of Approximation (RMSEA) to evaluate the model fit. A CFI > 0.90 was rated as acceptable and > 0.95 as good, a RMSEA < 0.6 was rated as good [58]. To evaluate the meaningfulness of changes of the model fit we used the change in the CFI (ΔCFI) because this index is proposed to be independent of overall model fit and sample size. A value of ΔCFI smaller than or equal to – 0.01 indicates that the null hypothesis of invariance should not be rejected [59]. Missings were dropped listwise.

We used linear and logistic regressions within SPSS for testing functional equivalence of the SDQ. SDQ total difficulties score or SDQ subscales and the sample subgroup (categorical variable with the German native group as reference group) were used as predictors, mental health diagnoses, use of mental health service or depressive symptoms as outcome variables. We tested for an interaction effect of group and SDQ scores indicating a different predictive power of the SDQ scores between the groups. Cross-sectional and longitudinal data was used.

## Results

### Descriptive statistics

The full sample (*N *= 11,812) used in this study comprises answers from *N *= 10,610 native German interview partners (*n *= 10560 first wave respondents and *n *= 50 second wave respondents), *N *= 534 Russian origin interview partners (*n *= 477 first wave respondents and *n *= 57 second wave respondents), and *N *= 668 Turkish origin interview partners (*n *= 620 first wave respondents and *n *= 48 second wave respondents). The three subsamples German native, Russian origin and Turkish origin parents differed from one another in some aspects. Whereas mothers were interview partners in most cases in the German native and in the Russian origin group (88.5% and 83.5%), this was only true for 57.9% in the Turkish origin group. All native German interview partners were born in Germany, but only 1.7% in the Russian origin group and 19.5% in the Turkish origin group. German native children had a higher SES than children of Russian origin, children of Turkish origin had the lowest SES. Children in the Turkish origin group were more often male (55.7%) and were slightly younger (M = 9.01) compared to the other two groups (Table 1). To avoid biasing effects due to age, gender and SES, for the measurement invariance analyses, two subsamples from the large German native group were drawn: In each strata (e.g. boys or high SES) a random sample was drawn with equal sample-sizes as in the corresponding strata in the Turkish/Russian origin group. After matching, there were no significant differences in age, gender and SES between the German native and the Turkish/Russian origin groups anymore and the groups were of equal sample size (matched German native sample for the Russian origin group *N *= 550, for the Turkish origin group *N *= 670).

**Table 1:** Sample characteristics

	Sample				
	Full sample	German natives	Russian origin	Turkish origin	Chi^2^-test/ ANOVA
*N*:	11,812	10,610	534	668	
Interview partner					
Mother	86.2%	88.5%	83.5%	57.9%	*χ*^2^ (4) = 506.09**
Father	8.9%	7.6%	8.4%	29.5%	
Both	4.9%	4.2%	8.1%	12.6%	
Interview partner born in Germany					
Yes	91.5%	100%	1.7%	19.5%	*χ*^2^ (6) = 10611.2**
No	7.5%	0%	90.3%	68.5%	
*Both parents*: one born in Germany	0.1%	0%	0.2%	2.6%	
*Both parents*: none born in Germany	0.9%	0%	7.9%	9.4%	
Gender of the child					
Male	51.0%	50.6%	52.8%	55.7%	*χ*^2^ (2) = 7.12*
Female	49.0%	49.4%	47.2%	44.3%	
Age of the child (3–17 years)					
*M*/*SD*	9.87/4.24	9.93/4.23	9.66/4.22	9.01/4.12	*F (2, 11809) *= 15.45**
Socio-economic status					
Low	13.9%	11.1%	27.7%	50.7%	*χ*^2^ (4) = 935.24**
Medium	61.2%	62.0%	62.9%	44.6%	
High	24.9%	26.9%	9.5%	4.7%	

*p < 0.05;**p < *0.01*

The SDQ response behaviour of the groups is displayed in Additional file 1.

### Differential item functioning

#### German native/Russian origin group

**Fig. 1:**
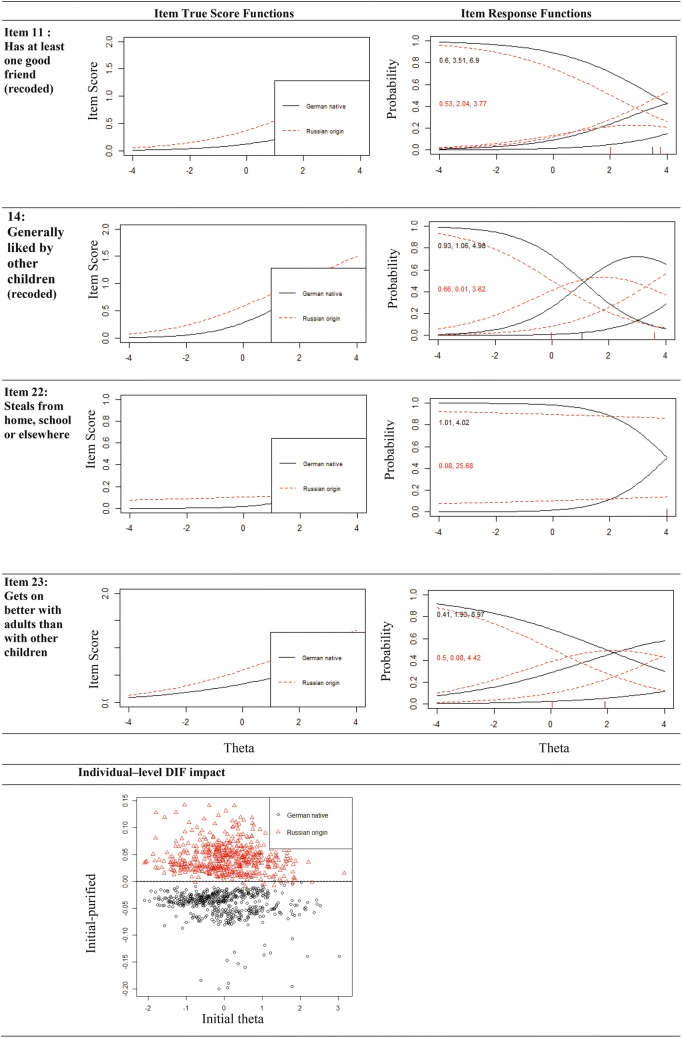
German native/Russian origin comparison: Item True Score Functions (item characteristic curves) and Item Response Functions of the items marked for DIF (numbers in Item Response Functions are category thresholds). The Item Response Functions display the probability of endorsing the item response options “not true” (0), “somewhat true” (1) or “certainly true” (2) as a function of the IRT theta score adjusted for DIF. Slope and category threshold values by group are displayed within the graphs. Individual–level DIF impact shows the difference in scores between using scores that ignore DIF and those that account for DIF. Positive values indicate that accounting for DIF led to lower SDQ scores, negative values indicate that accounting for DIF led to higher SDQ scores

When comparing the item-functioning of the items in the originally proposed 5-factor model with the logistic regression/IRT hybrid method (lordif), only Item 22 ‘Steals from home, school or elsewhere’ in the conduct problems scale was flagged for DIF (Δ*R*^2^1*,2 *= 0.0733 and Δ*R*^2^*2,3 *= 0.0868). When testing the total difficulties scale, four items were flagged: Item 22 ‘Steals from home, school or elsewhere’, Item 11 ‘Has at least one good friend’, Item 14 ‘Generally liked by other children’ and Item 23 ‘Gets on better with adults than with other children’ (Items 11, 14, 23 are from the peer problems subscale). Results are shown in Table 2 and Fig. 1. All the flagged items show uniform DIF, Item 22 also shows non-uniform DIF. For this item, the three answer categories were collapsed to two categories. Item thresholds and the Individual–level DIF impact figure indicate that accounting for DIF lead to lower total difficulties scores in Russian origin children and higher scores in German native children (Fig. 1).

**Table 2:** Differential item functioning in the German native and Russian origin subgroups

Item	Non-uniform DIF		Uniform DIF		
	χ^2^ 23	Δ*R*^2^	χ^2^ 12	Δ*R*^2^	Δβ12
11. Has at least one good friend	0.99	0.0339	0.00	0.0346	0.0346
14. Generally liked by other children	0.28	0.0006	0.00	0.0354	0.0401
22. Steals from home, school or elsewhere	0.00	0.0222	0.00	0.0526	0.0001
23. Gets on better with adults than with other children	0.21	0.0009	0.00	0.0234	0.0242

The MIMIC approach detected several items for DIF (Table 3). In the conduct problem scale, all items were detected for DIF, that is why a combined externalizing problems scale (conduct problems and hyperactivity) was tested. When taking into account the MIMIC-ES, the items 15 (‘Easily distracted, concentration wanders‘), 7 (‘Generally obedient, usually does what adults request’), 18 (‘Often lies or cheats’), 6 (‘Rather solitary, tends to play alone’), 19 (‘Picked on or bullied by other children’), 23 (‘Gets on better with adults than with other children’) show small DIF effects, item 5 (‘Often has temper tantrums or hot tempers’) shows a medium and item 22 (‘Steals from home, school or elsewhere’) shows a large DIF effect. Thus, only the items 22 and 23 show DIF within both analytic strategies.

**Table 3:** Items flagged for DIF and effect sizes within the MIMIC framework

German native/Russian origin group		German native/Turkish origin group	
Item and scale	MIMIC-ES	Item and scale	MIMIC-ES
Hyperactivity		Hyperactivity	
2	0.26	2	0.21
15	0.29	10	0.17
Externalizing problems		Internalizing problems	
2	0.23	11	0.46
15	0.30	14	0.14
5	0.53	23	0.70
7	0.31	3	0.18
18	0.45	13	0.05
22	1.90	16	0.06
Peer problems		Conduct problems	
6	0.36	22	0.25
19	0.45	18	0.08
23	0.41	7	0.13
Emotional problems		Emotional problems	
13	0.18	3	0.27
24	0.13	16	0.11
Prosocial behavior		Prosocial behavior	
9	0.17	4	0.07
17	0.23	20	0.12

#### German native/Turkish origin group

**Fig. 2:**
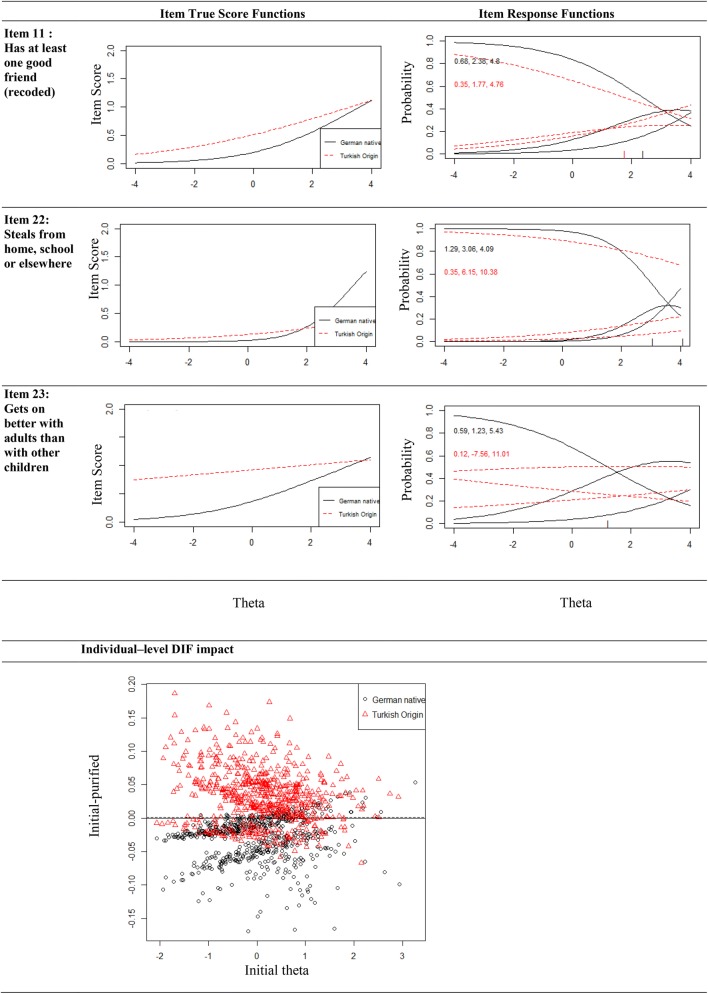
Item True Score Functions (Item Characteristic Curves) and Item Response Functions of the items marked for DIF in the German native/Turkish origin comparison (see Fig. 1 for explanatory comment)

Using the logistic regression/IRT hybrid method, item 22 from the conduct problems scale was marked for DIF. Within the peer problems scale, 4 of 5 items were marked for DIF. When testing the total difficulties scale, the items 22 (conduct problems), 11 and 23 (peer problems) were flagged for DIF (see Fig. 2 and Table 4). All items showed uniform DIF. Thresholds and the Individual–level DIF impact figure indicate that at lower levels of the trait, a purified scale without DIF items lead to a lower total difficulties score in Turkish origin children and a higher score in German native children. This effect seems to be less strong at higher levels of the trait.

**Table 4:** Differential Item Functioning in the German native and Turkish origin subgroups

Item	Non-uniform DIF		Uniform DIF		
	χ^2^ 23	Δ*R*^2^	χ^2^ 12	Δ*R*^2^	Δβ12
22. Steals from home, school or elsewhere	0.00	0.0187	0.00	0.0314	0.0051
23. Gets on better with adults than with other children	0.02	0.0025	0.00	0.0294	0.0063
11. Has at least one good friend	0.05	0.0073	0.00	0.0828	0.0473

The MIMIC method, when considering only DIF with an effect size above 0.3 (small effect) also results in the detection of item 11 (medium effect) and 23 (large effect; Table 3).

### Testing the configural model

In light of existing literature questioning the validity of the five factor solution and the described results above, indicating validity problems (in particular regarding the peer problems scale) the model fit of six different models were tested separately for the three subgroups: (1) A five factor model as proposed by Goodman [16]: hyperactivity, peer problems, conduct problems, emotional problems and prosocial behaviour, (2) a model with two additional higher order factors: internalizing behaviour (containing the subscales emotional problems and peer problems) and externalizing behaviour (containing the subscales hyperactivity and conduct problems), (3) a three factor model (internalizing behaviour, externalizing behaviour and prosocial behaviour), (4) a bifactor model with a general problem behaviour factor and the 5 factors proposed by Goodman [16], (5) a five factor model with an additional higher order general problem behaviour factor (containing the subscales hyperactivity, peer problems, conduct problems, emotional problems) and (6) a two factor model (general problem behaviour and prosocial behaviour). Because of the problems with the peer problems subscale, we additionally tested a model with a combined internalizing scale and the original three other scales (7).

The models were tested with and without the items detected for DIF within both methods in the previous analyses. Table 5 (with DIF items) and Table 6 (without DIF items) shows the fits of the models tested for each subgroup. The bifactor model (model 4) did not converge in any analysis. Only the original five factor model proposed by Goodman [16] reached an acceptable fit in the German natives group, but in none of the others. While the fits for the models were better in the Russian origin (CFI *M *= 0.78), than in the Turkish origin subgroup (CFI *M *= 0.72), in neither one they reached an acceptable fit.

**Table 5:** Model fit of configural models tested separately in the subgroups (with DIF items)

Sample	German native M1	German native M2	Russian origin	Turkish origin
Model, degrees of freedom, *p* value	5 factor model, df = 265, p < 0.001			
Chi^2^	603.172	603.172	692.881	1008.665
CFI	0.91	0.91	0.80	0.74
RMSEA (90% CI)	0.049 (0.44–0.55)	0.049 (0.44–0.55)	0.060 (0.054–0.065)	0.071 (0.067–0.76)
SRMR	0.088	0.088	0.095	0.101
Model, degrees of freedom, *p* value	5 factor model & higher order factors internalizing and externalizing problems, df = 268, p < 0.001			
Chi^2^	–^a^	633.641	691.862	1032.168
CFI	–	0.90	0.80	0.73
RMSEA (90% CI)	–	0.051 (0.046–0.056)	0.059 (0.054–0.064)	0.072 (0.067–0.076)
SRMR	–	0.091	0.095	0.103
Model, degrees of freedom, *p* value	5-factor model and general problem behaviour as higher-order factor, df = 270, p < 0.001			
Chi^2^	656.678	656.677836	684.840	1030.456
CFI	0.89716	0.897	0.802	0.73162212
RMSEA (90% CI)	0.052 (0.047–0.057)	0.052 (0.047–0.057)	0.058 (0.053–0.064)	0.071 (0.067–0.076)
SRMR	0.094	0.094	0.096	0.104
Model, degrees of freedom, *p* value	2 factor- model (general problem behaviour factor and prosocial behaviour factor), df = 274, p < 0.001			
Chi^2^	1110.233	1110.233	–^a^	1151.055
CFI	0.78	0.78	–	0.69
RMSEA (90% CI)	0.076 (0.072–0.081)	0.076 (0.072–0.081)	–	0.076 (0.072–0.081)
SRMR	0.122	0.122	–	0.109
Model, degrees of freedom, *p* value	3 factor model (internalizing & externalizing problem behaviour, prosocial behaviour), df = 265, p < 0.001			
Chi^2^	603.172	603.172	692.881	1008.665
CFI	0.91	0.91	0.80	0.74
RMSEA (90% CI)	0.049 (0.44–0.55)	0.049 (0.44–0.55)	0.060 (0.054–0.065)	0.071 (0.067–0.76)
SRMR	0.088	0.088	0.095	0.101
Model, degrees of freedom, *p* value	4 factor model (internalizing problem behaviour, conduct problems, hyperactivity, prosocial behaviour), df = 269, p < 0.001			
Chi^2^	699.267	699.267	736.841	1073.932
CFI	0.89	0.89	0.78	0.72
RMSEA (90% CI)	0.055 (0.050–0.060)	0.055 (0.050–0.060)	0.062 (0.057–0.067)	0.073 (0.069–0.078)
SRMR	0.097	0.097	0.097	0.105

*CI* confidence interval

^a^Model did not converge/was not identified

**Table 6:** Model fit of configural models tested separately in the subgroups without items marked for DIF

Sample	German native M1	German native M2	Russian origin	Turkish origin
Model, degrees of freedom, *p* value	5 factor model, df = 220, p < 0.001			
Chi^2^	524.275674	537.840014	652.941623	-^a^
CFI	0.91917157	0.91276914	0.79070152	–
RMSEA (90% CI)	0.051 (0.046–0.057)	0.052 (0.047–0.058)	0.065 (0.059–0.071)	–
SRMR	0.077	0.088	0.096	–
Model, degrees of freedom, *p* value	5 factor model & higher order factors internalizing and externalizing problems, df = 223, p < 0.001			
Chi^2^	–^a^	577.394	651.202	–^a^
CFI	–	0.903	0.793	–
RMSEA (90% CI)	–	0.055 (0.050–0.061)	0.064 (0.059–0.070)	–
SRMR	–	0.092	0.097	–
Model, degrees of freedom, *p* value	5-factor model and general problem behaviour as higher-order factor, df = 225, p < 0.001			
Chi^2^	579.197	594.173	641.429	825.560
CFI	0.906	0.899	0.799	0.779
RMSEA (90% CI)	0.055 (0.049–0.060)	0.056 (0.050–0.061)	0.063 (0.057–0.069)	0.069 (0.064–0.074)
SRMR	0.084	0.095	0.097	0.097
Model, degrees of freedom, *p* value	2 factor-model (general problem behaviour factor and prosocial behaviour factor), df = 229, p < 0.001			
Chi^2^	1033.461	989.836	882.058	935.999
CFI	0.786	0.791	0.684	0.739
RMSEA (90% CI)	0.082 (0.077–0.087)	0.08 (0.0745–0.085)	0.078 (0.072–0.084)	0.074 (0.069–0.079)
SRMR	0.113	0.120	0.111	0.104
Model, degrees of freedom, *p* value	3 factor model (internalizing & externalizing problem behaviour, prosocial behaviour), df = 227, p < 0.001			
Chi^2^	738.805	732.610	770.730	858.082
CFI	0.864	0.861	0.737	0.767
RMSEA (90% CI)	0.066 (0.060–0.071)	0.065 (0.060–0.070)	0.072 (0.066–0.077)	0.070 (0.065–0.076)
SRMR	0.096	0.105	0.104	0.098
Model, degrees of freedom, *p* value	4 factor model (internalizing problem behaviour, conduct problems, hyperactivity, prosocial behaviour), df = 224, p < 0.001			
Chi^2^	621.611886	611.2929	708.838157	845.806496
CFI	0.894	0.894	0.766	0.771
RMSEA (90% CI)	0.058 (0.053–0.064)	0.057 (0.052–0.063)	0.068 (0.063–0.074)	0.070 (0.065–0.075)
SRMR	0.088	0.095	0.098	0.097

*CI* confidence interval

^a^Model did not converge/was not identified

The deletion of the DIF items did not improve most of the model fits for the Russian origin group. The original five factor model did fit best to the Russian origin data (CFI = 0.79 without DIF items).

When allowing residual correlation within subscales and between positively worded items, the original five factor model showed an acceptable model fit in the Russian origin group (Chi^2^(210) = 402.121, CFI = 0.91, RMSEA(CI) = 0.044 (0.038–0.051), SRMR = 0.076) and in the German native group (matched sample; Chi^2^(210) = 432.913, CFI = 0.94, RMSEA(CI) = 0.044 (0.039–0.051), SRMR = 0.072).

Configural invariance was reached between the Russian origin and the German native group, but not weak invariance (Table 7). Thus, strong invariance was not tested.

**Table 7:** Measurement invariance: German native and Russian origin subgroup (5 factor model)

	Configural (df = 420)	Weak (df = 336)
Chi^2^	833.183	934.573
Δχ^2^		101.3898**
Chi^2^ German sample	420.373	443.885
Chi^2^ Russian sample	412.810	490.688
CFI	0.930	0.916
ΔCFI		0.014
TLI	0.92	0.90
RMSEA	0.045 (0.040–0.049)	0.048 (0.044–0.052)
ΔRMSEA		0.003
SRMR	0.074	0.081

**p < *0.01*

When deleting the items flagged for DIF in the previous analysis for each subgroup, most of the model fits improved for the Turkish origin group, while the first, second and the fifth model were not identified anymore. The seventh model without the DIF items reached the best fit (CFI = 0.77) in the Turkish origin group, but did not reach an acceptable fit even after allowing residual correlation within subscales and between positively worded items.

One reason for the insufficient fit might be the wording of the items. Since positively worded items tend to cluster together, some studies involved a positive construal factor to deal with the impact of wording [4, 60, 61]. However, including a common method factor might be problematic because it is impossible to estimate the exact effect of the common method variance without directly measuring the common source variable, possibly leading to a bias in the loadings of the other factors [62]. Because most of practitioners are using the subscales that describe problem behaviour only and not the prosocial behaviour subscale to screen for mental health problems anyway, we decided to test a configural model without the prosocial subscale items [31].

When allowing residual correlation within subscales and between positively worded items and neglecting the prosocial behaviour scale, an acceptable model fit (Chi^2^(122) = 302.201, CFI = 0.92, RMSEA(CI) = 0.051 (0.043–0.056), SRMR = 0.067) was reached. The same model also showed an acceptable/good fit in the German native group (matched sample; Chi^2^(122) = 261.949, CFI = 0.957, RMSEA(CI) = 0.047 (0.039–0.054), SRMR = 0.082). Testing invariance within the MGCFA framework revealed configural, metric and scalar invariance between the groups (Table 8).

**Table 8:** Measurement invariance: German native and Turkish origin subgroup (4 factor model)

	Configural (df = 244)	Weak (df = 341)	Strong (df = 358)
Chi^2^	596.5257	640.2125	674.3328
Δχ^2^		43.6868**	37.51
Chi^2^ German sample	292.209	299.781	318.411
Chi^2^ Turkish sample	304.317	340.432	355.921
CFI	0.949	0.945	0.942
ΔCFI		0.004	0.003
TLI	0.936	0.936	0.9357
RMSEA	0.0488 (0.0439–0.0538)	0.0493 (0.0445–0.0541)	0.049 (0.044–0.054)
ΔRMSEA		0.00	0.00
SRMR	0.069	0.0769	0.072

**p < *0.01*

### Additional analysis

We compared the total difficulties scores before and after exclusion of the DIF Items. In both analysis, problem behaviour was rated higher for children in the Turkish origin group and Russian origin group compared to the German native group, but the score difference was lower after excluding the DIF Items (Turkish origin/German native comparison original score: Δ*M *= 1.85; New score Δ*M* = 1.04; Russian origin/German native comparison original Score: Δ*M *= 1.16; New score Δ*M* = 0.90).

### Functional equivalence

We tested the predictive power of the SDQ total difficulties score within the first survey wave and the predictive power of the SDQ total difficulties score, hyperactivity subscale and emotional problems subscale in a longitudinal design using logistic and linear regression analysis with the German native group as reference group. The SDQ total difficulties scale and the emotional and hyperactivity subscales predicted mental health problems. However we did not find interaction effects for the SDQ scores and the group of origin (German, Russian, Turkish). Results are displayed in Table 9.

**Table 9:** Functional equivalence: linear and logistic regressions

	Unstandardized beta coefficient		
	SDQ-scale	Group	Interaction scale × group
Cross-sectional			
SDQ total difficulties score (wave 1)-contact to psychotherapist/psychologist/psychiatrist	0.181**		
Group Russian origin		− 0.536	0.134
Group Turkish origin		− 0.106	0.052
Longitudinal			
SDQ subscale hyperactivity (wave 1)-ADHD diagnosis (wave 2)	0.644**		
Group Russian origin		− 0.701	0.072
Group Turkish origin		− 1.00	0.093
SDQ subscale emotional problems (wave 1)-PHQ sumscore (wave 2) *Linear regression*	0.437**		
Group Russian origin		0.203	− 0.021
Group Turkish origin		2.48	− 0.224
SDQ total difficulties score (wave 1)-mental health disorder diagnosis (wave 2)	0.097**		
Group Russian origin		0.322	0.033
Group Turkish origin		− 0.186	0.876

**p < *0.01*

## Discussion

People from different cultural backgrounds may differ in the way they answer a questionnaire due to different response styles, reference groups or societal norms [5–7] and measures thus might be biased. Comparing measures across cultures requires cross-cultural comparability or methodologically spoken measurement invariance, which needs to be tested beforehand [63]. In the current study we examined the measurement invariance of the SDQ, a questionnaire measuring behavioural problems and strengths of children, for native German parents and parents of Russian and Turkish origin in Germany. To our knowledge, the current study is only the second to test measurement invariance in the parent report version of the SDQ between native parents and immigrant parents, the first one doing this with parents of Russian or Turkish origin and the first one in Germany. Items were detected for DIF in both the Russian origin/German native and the Turkish origin/German native comparisons. Whereas in the German native/Turkish origin analysis, the logistic regression/IRT hybrid method and the MIMIC model detection method flagged similar items for DIF, in the Russian origin/German native sample a lot more items were detected in the MIMIC framework. Moreover, comparing Russian origin and German native respondents by using the MGCFA framework to items not flagged for DIF, only configural invariance was reached. One reason for the unstable results could be a non-sufficient sample size in the Russian/German native comparison. Differing properties of the analyses might be another one: MIMIC analyses for DIF detection were found to work better in scales with a high percentage of DIF items [56] and with smaller sample sizes [64], but also seem to be vulnerable to detect false positives [65]. Only finding configural invariance moreover might be a result of deleting items only, if they were flagged for DIF in both preliminary analyses (MIMIC approach and logistic regression/IRT hybrid method). Thus DIF items remaining in the questionnaire led merely to configural invariance.

We replicated the five factor structure of the SDQ as proposed by Goodman [16] for the Russian origin, but not for the Turkish origin parents group. However, using a three factor structure (without the prosocial behaviour scale and with the peer problems and emotional problems scale combined to an internalizing problems scale), configural invariance (and also metric and scalar invariance) for the German native/Turkish origin comparison was found. Thus, given the original five factor structure of the SDQ, at least for the Turkish origin parents, it cannot be certain if the same underlying construct is measured compared to the German native parents.

The five factor structure of the SDQ was already questioned by other studies: Mellor and Stokes [66] evaluated the five factor structure as inadequate and several studies found a better fit for a three factor solution [29, 67]. A higher order factor model or a bifactor model (as proposed in [46, 68, 69]) did not reach an acceptable fit in our analyses. Some studies suspected the prosocial subscale to be problematic (e.g. [31]). This might be a result of the combination of the positively worded prosocial subscale with positively worded (reversed) items in the problem subscales, because the positively worded items tend to cluster together [59]. Essau et al. [70] chose another solution and removed the reversed items, afterwards they found an improved fit. We also found acceptable model fits in the immigrant groups only after allowing positively worded item residuals to correlate.

Whereas research about the child rearing values in Russian immigrants in Germany is very scarce, some studies compared German native with Turkish origin parents. Parents of Turkish origin in Germany were more likely than German native parents to expect close family relations, mutual support in the family, obedience and being well-mannered and they were less likely to value autonomy or self-control in their children [13–15]. First and second generation mothers had quite similar socialization goals, second-generation mothers still highly valued their traditional Turkish socialization patterns [71]. Unfortunately, we do not have the data necessary to investigate the underlying reasons for the DIF and the missing equivalence of the factor structure in our study. However, because we matched the samples according to SES, age and gender of the child, none of these factors is apparently the reason for the lack of invariance when using the whole set of items. Hypotheses to be tested in future research could be, that the item detected for DIF from the original peer problems subscale ‘Gets on better with adults than with other children’ is understood as a part of family closeness or obedience and thus does not belong to a peer problem construct in Turkish origin and Russian origin parents. Or that the item ‘Steals from home, school or elsewhere’ could be biased by social desirability in the Russian and Turkish origin subgroup less strongly than in the German native group. The peer problems subscale, to which two of the three items detected for DIF belong, was also found to have a low internal consistency in other studies, Husky et al. [43] recommend to exclude the scale when one wants to predict internalizing mental health disorders.

Despite the need of cautiousness when comparing SDQ results, our study supports the usability of the SDQ as a screening tool in groups of different cultural origin. We did not find a difference in the predictive power of SDQ scores between the groups (concerning depressive symptoms, ADHD and mental disorders in general).

With regard to limitations of our study, first of all, the sample size was maybe too small to detect all DIF items or to gain stable results in the Russian origin sample. We could not cross-validate the results with data from the second available survey wave, because the immigrant sample was too small for a separate analysis. Instead we added respondents from this wave to the sample of the first wave to increase power. The missing representativeness of the second sample might have affected our longitudinal functional equivalence analysis. Additionally, we do not have objective data to evaluate the *real* behavioural problems of the children; the report of depressive symptoms or the existence of an ADHD diagnosis are also possibly biased, the former by response styles and the latter e.g. by different health care utilization behaviour. Accordingly, other measures, like observational data or the use of vignettes, might give more insight into the equivalence of the SDQ results. It would also be interesting to test measurement invariance between immigrant groups and the population in the countries of origin.

However, our study also has strong implications. It is not clear if differences in the level of behavioural problems between immigrant and native German children (e.g. in the studies [18–21]) are actual differences or consequences of lacking measurement invariance. Our results are in line with results of other studies, that found a lack of measurement invariance in SDQ self- report data of adolescents of different cultural origins (e.g. [42, 45]). It is worth mentioning that we already did not use very strict criteria when testing DIF and model fit: We reported MIMIC-ES instead of just significant effects and used two approaches to validate the results. In the analysis of model fit, we allowed residual correlations and accepted CFI parameters of 0.90 instead of 0.95.

For both immigrant groups, the comparison with the German native group revealed smaller differences in the total difficulties scale after exclusion of DIF items. Thus, it is possible that the use of original questionnaire leads to an overestimation of differences between native and immigrant groups. This is relevant when the SDQ is used to examine if immigrant children are at special risk for mental illness, e.g. for prevention planning. We only tested equivalence in two immigrant groups, but it is highly possible that the issue also affects the measurement in immigrants from other countries of origin. The limited amount of research in African countries [72, 73] and the research conducted with refugee children [74] also indicate to be careful when using the SDQ.

## Conclusions

Summarizing, our results indicate that one has to be cautious using the SDQ to compare behavioural problems in groups of different cultural origins. It is not advisable to directly compare the scores of the original scales. Measurement invariance should always be tested before drawing conclusions. If there is a lack of invariance, adapted scales or latent models should be used. However, the SDQ still seems to be a valuable instrument for the screening for mental disorders in native children as well as in children of immigrants.

## Supplementary information

**Additional file 1.** Parental SDQ response behaviour in the three groups (reports on their child).

## Data Availability

The data that support the findings of this study are available from the RKI but restrictions apply to the availability of these data, which were used under license for the current study, and so are not publicly available. Data are however available from the RKI upon reasonable request.

## Abbreviations

SDQ: Strengths and Difficulties questionnaire
KiGGS: German health interview and examination survey for children and adolescents
RKI: Robert Koch Institute
SES: Socioeconomic Status
ADHD: Attention Deficit Hyperactivity Disorder
DIF: Differential Item Functioning
IRT: Item response theory
MIMIC-ES: The multiple indicators, multiple causes model effect size
CFI: Comparative Fit Index
RMSEA: Root Mean Square Error of Approximation
