# Supplementary material for: Measuring children’s emotional and behavioural problems: are SDQ parent reports from native and immigrant parents comparable?
Source: Child Adolesc Psychiatry Ment Health. 2019 Nov 28;13:46. doi: 10.1186/s13034-019-0306-z (PMC6882192; doi:10.1186/s13034-019-0306-z)
Supplement: Supplementary file 1 — Additional file 1. Parental SDQ response behaviour in the three groups (reports on their child). [file 13034_2019_306_MOESM1_ESM.docx]

Additional file 1

Parental SDQ response behaviour in the three groups (reports on their child)

|  | SDQ Item | | German native parents | | | Russian origin parents | Turkish origin parents |
| --- | --- | --- | --- | --- | --- | --- | --- |
|  | Sample size^1^ | | a) Complete Sample *N*=10595  b) Matched Sample 1^2^ *N*=550  c) Matched Sample 2^2^ *N*=670 | | | *N*=534 | *N*=668 |
| No. | Content | | a) | b) | c) |  |  |
| 1 | Considerate of other  people's feelings | Not true | 3.1 % | 4.0 % | 3.9 % | 7.6 % | 8.4 %, |
|  | Somewhat true | | 55.4 % | 63.7 % | 56.8 % | 49.5 % | 48.9% |
|  | Certainly true | | 40.8 % | 32.3 % | 39.3 % | 42.9 % | 42.7% |
| 2 | Restless, overactive,  cannot stay still for long | Not true | 61.6 % | 59.8 % | 55.6 % | 46.4 % | 41.4 % |
|  | Somewhat true | | 29.1 % | 29.4 % | 32.2 % | 34.9 % | 35.4 % |
|  | Certainly true | | 8.7 % | 10.8 % | 12.2 % | 18.7 % | 23.2 % |
| 3 | Often complains of  headaches, stomach-  aches or sickness | Not true | 65.7 % | 64.0 % | 61.2 % | 67.4 % | 68.8 % |
|  | Somewhat true | | 28.7 % | 30.3 % | 31.5 % | 26.2 % | 26.2 % |
|  | Certainly true | | 5.0 % | 5.7 % | 7.3 % | 6.4 % | 5.0 % |
| 4 | Shares readily with other children (treats, toys, pencils etc.) | Not true | 2.9 % | 4.0 % | 3.1 % | 2.1 % | 3.3 % |
|  | Somewhat true | | 41.1 % | 42.0 % | 41.9 % | 36.1 % | 36.7 % |
|  | Certainly true | | 55.7 % | 54.0 % | 54.9 % | 61.8 % | 60.0 % |
| 5 | Often has temper tantrums or hot tempers | Not true | 51.3 % | 47.4 % | 47.6 % | 57.7 % | 49.5 % |
|  | Somewhat true | | 40.2 % | 43.2 % | 43.4 % | 35.7 % | 40.2 % |
|  | Certainly true | | 8.4 % | 9.5 % | 9.1 % | 6.6 % | 10.3 % |
| 6 | Rather solitary, tends to play alone | Not true | 72.5 % | 71.2 % | 69.7 % | 66.4 % | 68.4 % |
|  | Somewhat true | | 23.1 % | 25.5 % | 25.4 % | 26.0 % | 24.4 % |
|  | Certainly true | | 4.1 % | 3.3 % | 4.9 % | 7.5 % | 7.2 % |
| 7 | Generally obedient, usually does what adults request | Not true | 4.9 % | 5.1 % | 6.1 % | 9.4 % | 11.2 % |
|  | Somewhat true | | 59.5 % | 63.6 % | 58.1 % | 64.4 % | 59.7 % |
|  | Certainly true | | 35.4 % | 31.3 % | 35.8 % | 26.3 % | 29.1 % |
| 8 | Many worries, often seems worried | Not true | 77.9 % | 72.6 % | 75.6 % | 71.0 % | 72.5 % |
|  | Somewhat true | | 19.0 % | 23.0 % | 21.5 % | 24.9 % | 22.0 % |
|  | Certainly true | | 2.6 % | 4.4 % | 2.8 % | 4.2 % | 5.5 % |
| 9 | Helpful if someone is hurt, upset or feeling ill | Not true | 1.9 % | 2.0 % | 2.4 % | 2.8 % | 3.8 % |
|  | Somewhat true | | 21.7 % | 25.5 % | 24.4 % | 26.3 % | 25.5 % |
|  | Certainly true | | 76.3 % | 72.5 % | 73.2 % | 70.9 % | 70.7 % |
| 10 | Constantly fidgeting or squirming | Not true | 68.9 % | 65.1 % | 62.9 % | 61.4 % | 52.5 % |
|  | Somewhat true | | 25.1 % | 27.5 % | 29.1 % | 29.1 % | 30.9 % |
|  | Certainly true | | 5.7 % | 7.5 % | 7.9 % | 9.6 % | 16.6 % |
| 11 | Has at least one good friend | Not true | 3.4 % | 1.8 % | 4.3 % | 13.2 % | 16.9 % |
|  | Somewhat true | | 10.0 % | 10.2 % | 13.6 % | 13.8 % | 19.2 % |
|  | Certainly true | | 86.4 % | 88,0 % | 82.0 % | 73.0 % | 63.9 % |
| 12 | Often fights with other children or bullies them | Not true | 79.5 % | 74.2 % | 74.1 % | 69.7 % | 70.3 % |
|  | Somewhat true | | 18.6 % | 24.2 % | 23.6 % | 28.4 % | 25.5 % |
|  | Certainly true | | 1.7 % | 1.6 % | 2.2 % | 1.9 % | 4.2 % |
| 13 | Often unhappy, down-hearted or tearful | Not true | 84.3 % | 82.0 % | 79.5 % | 75.0 % | 73.3 % |
|  | Somewhat true | | 13.6 % | 16.4 % | 18.8 % | 21.2 % | 21.2 % |
|  | Certainly true | | 2.0 % | 1.6 % | 1.6 % | 3.9 % | 5.6 % |
| 14 | Generally liked by other children | Not true | 2.0 % | 1.5 % | 2.1 % | 10.0 % | 9.4 % |
|  | Somewhat true | | 25.0 % | 28.2 % | 28.6 % | 40.6 % | 30.6 % |
|  | Certainly true | | 72.8 % | 70.3 % | 69.3 % | 49.4 % | 60.0 % |
| 15 | Easily distracted, concentration wanders | Not true | 38.6 % | 31.6 % | 32.0 % | 38.3 % | 32.0 % |
|  | Somewhat true | | 47.3 % | 51.6 % | 51.6 % | 49.2 % | 51.4 % |
|  | Certainly true | | 13.9 % | 16.7 % | 16.4 % | 12.4 % | 16.6 % |
| 16 | Nervous or clingy in new situations, easily loses confidence | Not true | 52.9 % | 48.5 % | 48.5 % | 53.4 % | 50.6 % |
|  | Somewhat true | | 37.3 % | 40.1 % | 39.3 % | 40.3 % | 38.4 % |
|  | Certainly true | | 9.5 % | 11.5 % | 12.2 % | 6.4 % | 11.0 % |
| 17 | Kind to younger children | Not true | 2.3 % | 1.8 % | 2.7 % | 2.6 % | 4.4 % |
|  | Somewhat true | | 19.1 % | 18.4 % | 19.6 % | 20.5 % | 20.0 % |
|  | Certainly true | | 78.4 % | 79.7 % | 77.7 % | 76.9 % | 75.6 % |
| 18 | Often lies or cheats | Not true | 67.4 % | 60.4 % | 60.3 % | 66.9 % | 66.1 % |
|  | Somewhat true | | 29.1 % | 35.9 % | 36.1 % | 31.6 % | 30.9 % |
|  | Certainly true | | 3.3 % | 3.6 % | 3.6 % | 1.5 % | 3.0 % |
| 19 | Picked on or bullied by other children | Not true | 80.8 % | 76.9 % | 78.7 % | 73.6 % | 78.5 % |
|  | Somewhat true | | 16.1 % | 18.7 % | 18.0 % | 22.2 % | 18.1 % |
|  | Certainly true | | 2.7 % | 4.4 % | 3.3 % | 4.2 % | 3.1 % |
| 20 | Often volunteers to help others (parents, teachers, other children) | Not true | 4.0 % | 2.2 % | 4.3 % | 6.0 % | 5.9 % |
|  | Somewhat true | | 52.7 % | 55.4 % | 48.7 % | 43.2 % | 35.5 % |
|  | Certainly true | | 43.2 % | 42.4 % | 46.9 % | 50.8 % | 58.6 % |
| 21 | Thinks things out before acting | Not true | 4.8 % | 5.9 % | 6.7 % | 11.1 % | 12.0 % |
|  | Somewhat true | | 62.6 % | 68.9 % | 66.2 % | 63.4 % | 56.8 % |
|  | Certainly true | | 32.2 % | 25.2 % | 27.1 % | 25.6 % | 31.3 % |
| 22 | Steals from home, school or elsewhere | Not true | 97.0 % | 97.3 % | 96.4 % | 89.6 % | 89.0 % |
|  | Somewhat true | | 2.0 % | 2.2 % | 2.5 % | 8.5 % | 8.2 % |
|  | Certainly true | | 0.8 % | 0.5 % | 1.0 % | 1.9 % | 2.7 % |
| 23 | Gets on better with adults than with other children | Not true | 71.6 % | 68.2 % | 67.0 % | 50.8 % | 28.5 % |
|  | Somewhat true | | 24.7 % | 29.0 % | 28.7 % | 38.3 % | 50.2 % |
|  | Certainly true | | 3.4 % | 2.7 % | 4.4 % | 11.0 % | 21.2 % |
| 24 | Many fears, easily scared | Not true | 69.4 % | 68.7 % | 66.5 % | 63.3 % | 61.7 % |
|  | Somewhat true | | 26.2 % | 26.0 % | 28.0 % | 27.4 % | 30.9 % |
|  | Certainly true | | 4.3 % | 5.3 % | 5.5 % | 9.4 % | 7.4 % |
| 25 | Sees tasks through to the end, good attention span | Not true | 8.9 % | 10.6 % | 10.0 % | 10.2 % | 12.2 % |
|  | Somewhat true | | 54.1 % | 60.0 % | 59.6 % | 62.6 % | 52.0 % |
|  | Certainly true | | 36.8 % | 29.4 % | 30.4 % | 27.3 % | 35.9 % |
| ^1^Sample sizes reflect whole samples, there are small deviations in sample size in the single items  ^2^German samples matched in SES, age and gender of the child to the Russian origin sample (matched sample 1) and the Turkish origin sample (matched sample 2) | | | | | | | |
